# Editorial: Novel anti-cancer agents targeting triple-negative breast cancer

**DOI:** 10.3389/fonc.2026.1906771

**Published:** 2026-09-02

**Authors:** Tong Zhu, Baifang Ding, Zhengnan Zhu, Jiawen Bu, Tianyu Guo, Zhipeng Jin, Xudong Zhu, Yefu Liu

**Affiliations:** 1 Department of Breast Surgery, Panjin Central Hospital, Panjin, Liaoning, China; 2 Department of Pediatrics, Shengjing Hospital of China Medical University, Shenyang, Liaoning, China; 3 Department of Colorectal Surgery, Shengjing Hospital of China Medical University, Shenyang, Liaoning, China; 4 Department of Hepatopancreatobiliary Surgery, Cancer Hospital of Dalian University of Technology, Liaoning Cancer Hospital and Institute, Shenyang, Liaoning, China; 5 Liaoning Provincial Key Laboratory of Precision Medicine for Malignant Tumors, Cancer Hospital of Dalian University of Technology, Liaoning Cancer Hospital and Institute, Shenyang, Liaoning, China; 6 Markey Cancer Center, University of Kentucky, Lexington, KY, United States

**Keywords:** ADCs, anti‑cancer agents, case report, editorial, triple‑negative breast cancer

Triple-negative breast cancer (TNBC) accounts for approximately 15%–20% of all breast cancer cases (1–3). Due to the lack of estrogen receptor (ER), progesterone receptor (PR), and human epidermal growth factor receptor 2 (HER2) expression, TNBC lacks well-defined and effective targeted therapeutic options (4). For decades, cytotoxic chemotherapy represented the mainstay of systemic treatment. In recent years, with the advancing research on immunotherapy, antibody-drug conjugates (ADCs), and molecular subtyping, the treatment landscape for TNBC has been undergoing significant changes. Nevertheless, challenges such as drug resistance, metastasis, and high tumor heterogeneity of TNBC remain major obstacles in current clinical practice (5). Consequently, deriving therapeutic strategies from real-world evidence, accelerating the translation of basic research into clinical applications and identifying novel therapeutic targets have become critical directions in the area of TNBC research.

This Research Topic aggregates a comprehensive array of studies spanning real-world clinical evidence, targeted ADC development, and fundamental mechanistic insights. It offers a comprehensive overview of the current therapeutic landscape of TNBC, as well as the evolving trends and future directions of anti-TNBC strategies (**Figure 1**).

**Figure 1:**
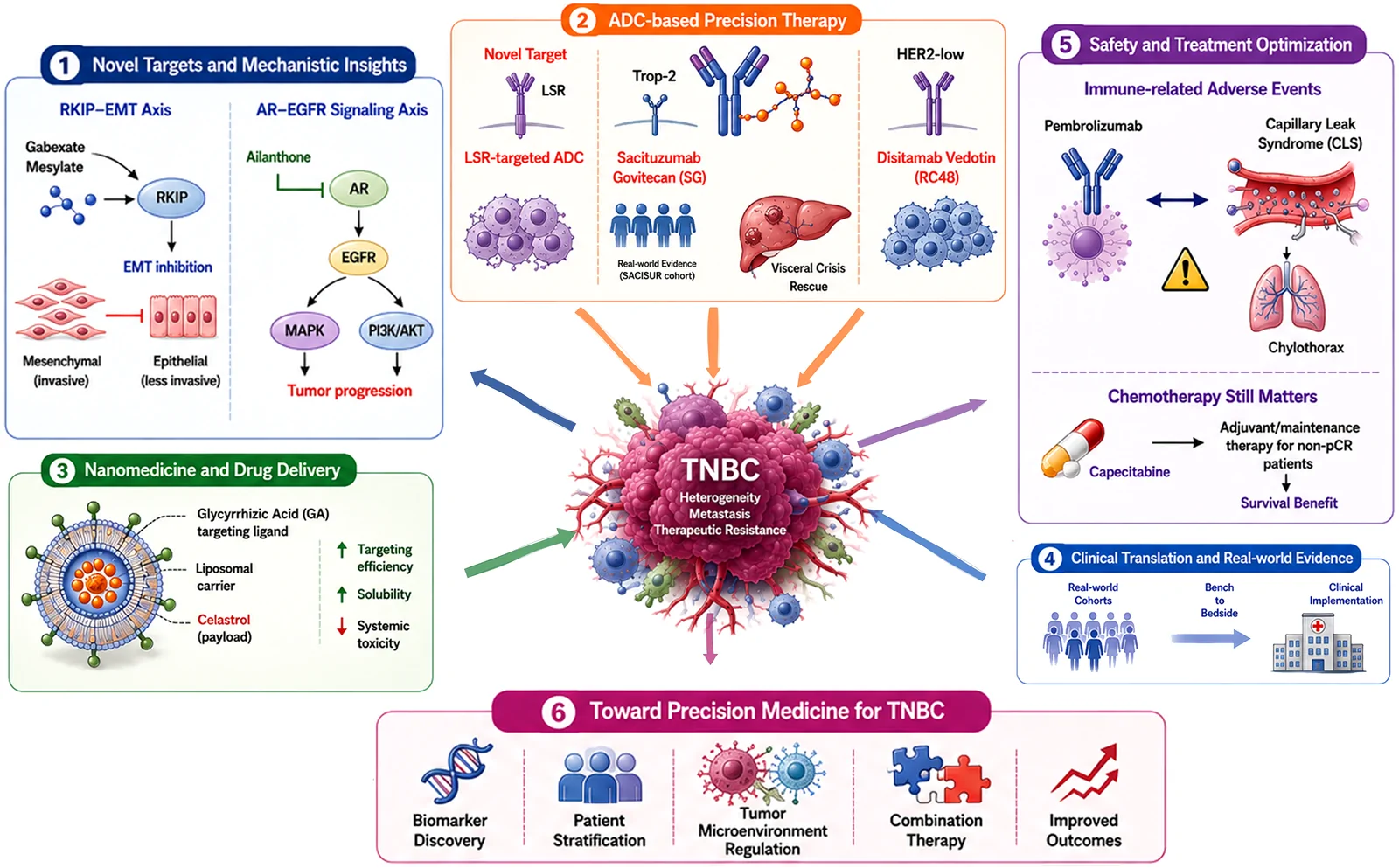
This figure summarizes the research findings compiled in this Research Topic. By integrating novel target exploration, ADCs development, optimized nanomedicine drug delivery technology, real-world clinical validation, therapeutic safety management and combination strategies with conventional chemotherapy, it systematically elaborates that the therapeutic paradigm for TNBC is gradually shifting from traditional empirical cytotoxic chemotherapy to tumor biology-oriented precision medicine.

## Reassessing standard-of-care: insights from real-world evidence

Before introducing additional therapeutic innovations, it is necessary to reconsider whether established treatment strategies provide consistent benefit across the biologically heterogeneous TNBC population. Patients with residual disease after neoadjuvant chemotherapy represent a particularly high-risk subgroup, for whom the CREATE-X trial established adjuvant capecitabine as a standard therapeutic approach. However, in a real-world analysis by Godoy-Ortiz et al., involving 312 patients from two Spanish institutions, the clinical benefit of adjuvant capecitabine appeared more limited than expected. Although patients achieving pathological complete response (pCR) experienced substantially improved outcomes, those with residual disease did not demonstrate a significant improvement in disease-free survival or overall survival compared with observation alone (*P* = 0.94 and *P* = 0.34, respectively). These findings are consistent with observations from the CIBOMA trial, which suggested that capecitabine efficacy may vary according to molecular subtype, with potential benefit restricted to non-basal-like TNBC. Collectively, therapeutic decisions based solely on clinical classification may fail to capture the underlying biological diversity of TNBC. Even established regimens require molecular reassessment to define which patients are most likely to benefit. This principle provides an important foundation for determining the optimal clinical context in which ADCs and other emerging therapies should be incorporated.

## ADCs redefining the therapeutic landscape: from trop-2 to HER2-low biology

Among the recent advances transforming TNBC treatment, ADCs represent one of the most clinically significant developments. Sacituzumab govitecan (SG), an anti-Trop-2 ADC, has become an established treatment option for patients with previously treated metastatic TNBC following the results of the ASCENT trial. In the SACISUR cohort, Falcon-Gonzalez et al. evaluated 159 patients from Southern Spain and reported real-world outcomes comparable to those observed in ASCENT, with median rwPFS and rwOS of 4.6 and 10.9 months, respectively. Notably, 13.8% of patients had brain metastases, which experienced poorer outcomes (rwPFS, 2.3 months), but the presence of measurable clinical activity suggests that SG may retain therapeutic potential in patients with central nervous system involvement. Therefore, emerging real-world evidence is extending our understanding of SG activity beyond the populations typically enrolled in randomized clinical trials.

Beyond conventional trial populations, preliminary evidence suggests that SG may also have activity in high-risk clinical presentations such as hepatic visceral crisis (HVC). Ye et al. reported a case of a patient with TNBC characterized by diffuse hepatic metastases and rapidly worsening liver function who achieved substantial regression of liver lesions after two cycles of SG, with a PFS of 16.3 months. Although limited to a single case, this observation provides a hypothesis-generating finding that ADC-based therapy may warrant consideration even in patients with severe tumor burden and restricted therapeutic options.

The expanding role of ADCs is further illustrated by the emergence of the HER2-low paradigm, which has challenged the traditional binary classification between HER2-positive and HER2-negative breast cancer. Wu et al. described a heavily pretreated patient with HER2-low metastatic breast cancer who achieved a favorable response to disitamab vedotin (RC-48), an anti-HER2 ADC developed in China. More importantly, serial evaluation revealed dynamic changes in HER2 expression throughout disease evolution: HER2-low at initial diagnosis, HER2–0 at first recurrence, and HER2-low again at subsequent progression. This expression shift highlights a fundamental aspect of tumor; biomarker expression can evolve under therapeutic pressure. Clinically, these findings support the consideration of repeat biomarker assessment at disease progression, as HER2 status may represent a dynamic therapeutic determinant rather than a fixed classification. Such an approach may help identify additional patients who could benefit from ADC strategies during the course of their disease.

## Balancing efficacy and safety: recognizing rare but serious toxicities

The expanding use of immunotherapy and ADCs in TNBC underscores the need to maintain a parallel focus on safety, particularly for rare but potentially life-threatening adverse events. Benkalfate et al. described a case of pembrolizumab-associated capillary leak syndrome (CLS) accompanied by chylothorax in a patient receiving immunotherapy for early-stage TNBC. Following two cycles of adjuvant pembrolizumab, the patient developed diffuse subcutaneous edema, bilateral pleural effusions, and ascites despite having previously tolerated neoadjuvant exposure. A comprehensive evaluation excluded infectious, malignant, cardiac, and autoimmune causes, while pleural biopsy showed no evidence of malignant or inflammatory infiltration, suggesting an endothelial dysfunction–mediated mechanism. Rapid clinical improvement following corticosteroid therapy further supported the diagnosis. Although rare, such events highlight a critical consideration as immune checkpoint inhibitors move increasingly into curative-intent settings. Therapeutic success must be accompanied by vigilant toxicity surveillance. Early recognition, systematic evaluation, and timely intervention remain essential for minimizing the impact of severe immune-related adverse events.

## Beyond current targets: expanding the biological and therapeutic landscape of TNBC

Despite the clinical success of existing ADCs, the molecular heterogeneity of TNBC suggests that no single target will be sufficient to address the full spectrum of disease biology. Expanding the repertoire of actionable targets therefore remains a critical objective. Zhou et al. identified lipolysis-stimulated lipoprotein receptor (LSR) as a potential ADC target, demonstrating its overexpression in more than 60% of TNBC samples and its association with adverse clinical features. An LSR-directed ADC incorporating a DM1 payload showed substantial antitumor activity in animal models, including marked tumor reduction or complete regression, without apparent toxicity across major organs. Although these findings remain preclinical, they provide a rationale for further investigation of LSR as a candidate target beyond currently established ADC strategies.

Beyond surface antigens, tumor signaling networks represent another avenue for therapeutic exploration. The androgen receptor (AR) has emerged as a potential vulnerability in selected TNBC subsets, although its biological role remains incompletely defined. Caceres et al. demonstrated that AR promotes TNBC cell proliferation through EGFR upregulation and MAPK/PI3K pathway activation, while attenuating ERβ-mediated anti-proliferative effects. Their evaluation of AR antagonists identified ailanthone as a potent inhibitor of AR signaling, including suppression of AR full length and AR-V7, accompanied by reduced Src expression and impaired cellular migration.

In parallel, addressing metastatic dissemination remains essential for improving TNBC outcomes. Deserti et al. investigated gabexate mesylate (GM), an established protease inhibitor, and found that it restored expression of Raf kinase inhibitor protein (RKIP), thereby attenuating MAPK and NF-κB signaling and reversing epithelial–mesenchymal transition (EMT). The resulting reduction in migration, invasion, and MMP-2/MMP-9 activity suggests a potential role for drug repurposing approaches in targeting metastatic mechanisms. However, further validation is required to determine whether these preclinical observations can translate into clinically meaningful benefit.

Complementing target discovery and pathway modulation, advances in drug delivery may provide additional opportunities to improve therapeutic windows. Zhang et al. developed glycyrrhizic acid-modified liposomes for delivery of celastrol, a bioactive compound limited by systemic toxicity. This nanotechnology-based formulation enhanced tumor uptake, improved drug stability and loading capacity, and increased cytotoxic activity against TNBC cells while reducing effects on normal mammary epithelial cells. Such approaches exemplify how delivery engineering can transform biologically active but clinically constrained molecules into more viable therapeutic candidates.

## Conclusions and perspectives

Overall, the studies included in this Research Topic illustrate a significant shift occurring in the field of TNBC treatment. This shift is moving away from traditional empirical cytotoxic therapy toward a precision medicine approach based on the biological characteristics of the tumor. Whether through the discovery of novel molecular targets, the development of ADCs and nano delivery systems, or the accumulation of real-world clinical evidence, these studies indeed reflect the refinement of treatment strategies for TNBC. In the future, with further advances in patient stratification, biomarker screening, tumor microenvironment regulation, and combination therapy strategies, precision treatment of TNBC is expected to deliver more individualized and sustainable clinical benefits.
